# A Review of Microwave-Assisted Reactions for Biodiesel Production

**DOI:** 10.3390/bioengineering4020057

**Published:** 2017-06-15

**Authors:** Saifuddin Nomanbhay, Mei Yin Ong

**Affiliations:** Institute of Sustainable Energy, Universiti Tenaga Nasional, 43000 Kajang, Selangor, Malaysia; me089475@hotmail.com

**Keywords:** microwave, sustainability, biodiesel, non-thermal effect, non-catalytic transesterification

## Abstract

The conversion of biomass into chemicals and biofuels is an active research area as trends move to replace fossil fuels with renewable resources due to society’s increased concern towards sustainability. In this context, microwave processing has emerged as a tool in organic synthesis and plays an important role in developing a more sustainable world. Integration of processing methods with microwave irradiation has resulted in a great reduction in the time required for many processes, while the reaction efficiencies have been increased markedly. Microwave processing produces a higher yield with a cleaner profile in comparison to other methods. The microwave processing is reported to be a better heating method than the conventional methods due to its unique thermal and non-thermal effects. This paper provides an insight into the theoretical aspects of microwave irradiation practices and highlights the importance of microwave processing. The potential of the microwave technology to accomplish superior outcomes over the conventional methods in biodiesel production is presented. A green process for biodiesel production using a non-catalytic method is still new and very costly because of the supercritical condition requirement. Hence, non-catalytic biodiesel conversion under ambient pressure using microwave technology must be developed, as the energy utilization for microwave-based biodiesel synthesis is reported to be lower and cost-effective.

## 1. Introduction

The microwave processing technology has revolutionized organic synthesis, and this technique has gained huge interest as a tool for synthesis and process development. In the last 30 years we have witnessed climate change—temperatures rising, weather becoming more erratic, and glaciers continuing to melt due to a higher level of greenhouse gas (GHG) emissions. Many integrated assessments have shown that increasing anthropogenic GHG emissions are largely caused by the use of fossil fuels, and hence there is a need for a range of renewable technology options to meet stringent global warming targets (e.g., keeping CO_2_ concentration below 440 ppm by 2050) [1]. The major component of GHGs is carbon dioxide, and there is a global concern about reducing carbon emissions. For thousands of years, the global carbon supply was essentially stable, as natural processes removed as much carbon as they released. Modern human activity—burning of fossil fuels, deforestation, and intensive agriculture—has led to huge quantities of carbon dioxide and other GHG emissions [2]. In order to rectify these problems, the dependency on fossil fuels should be scaled down. Switching to renewable energy will play a very significant role in combating climate change. Microwave technology can be used as an enabling tool to enhance reaction yield and selectivity for renewable energy processes. The need for sustainable development and green processing technologies will enhance interdisciplinary efforts and put microwave-based processes in the forefront.

Renewable energy is energy generated from natural resources—such as sunlight, wind, rain, tides, and geothermal heat—that are renewable (naturally replenished). Renewable energy technologies range from solar power, wind power, hydroelectricity/micro-hydro, biomass, and biofuels. This energy cannot be exhausted and is constantly renewed. In recent years, there has been a great interest in stimulating the use of bioenergy and biomaterials in order to reduce the GHG emissions associated with using fossil fuels. On the other hand, the biggest drawback of renewable energy is its relatively high cost compared to the conventional fossil-fuel energy sources. Additionally, with the current renewable energy technology, the amount of renewable electricity generated will not be as large as that produced by the conventional fossil fuels. Furthermore, the supply of renewable energy sources is not reliable, as most of them are based on the weather, which is unpredictable. Since 2011, the cost of renewable energy generation and energy storage equipment has fallen massively. Large reductions have occurred in the cost of lithium-ion batteries and related systems for energy storage. Cost reductions also come from economies of scale due to a large increase in production by China and other countries. Most importantly, advances in technology will keep prices falling after scale economies have been exhausted [3,4,5]. Any country with rich renewable energy resources and technological advancements will become a low-cost location for energy supplies in a low-carbon world economy. It will also make it an economically superior location for energy-intensive processing and manufacturing activity.

Recently, the rapid increase in chemical reaction rates by using microwave irradiation has attracted the attention of almost all fields of researchers, especially those in the chemistry area. Early researchers [6,7] reported that the microwave-assisted reaction rates increased, by a factor of 5, to 1000, when compared to the conventional reactions. The whole scientific and industrial community is interested with the application of microwave irradiation, not only due to its enhancement in the reaction rates, but also for its ability to revolutionize the chemical reactions to be performed with unexplainable results. In addition to these, there are several advantages of microwave processing [8,9,10], such as rapid heating, lower relative energy consumption, environmental friendliness, higher production yield, controllable processing, shorter processing time, and quality and properties improvement.

In this paper, some basic information on microwaves, such as their heating mechanisms and characteristics, are discussed in detail. Additionally, an overview of thermal and non-thermal effects of microwave heating is also presented. Furthermore, the application of microwave energy in various fields is also mentioned, particularly in biodiesel production, as biodiesel is a renewable fuel in liquid state, which provides advantages over the other renewable energies as most of the industrial areas and transportation use liquid fuel to operate. The purpose of this review paper is to provide a basic understanding regarding microwave energy and microwave-assisted biodiesel production for novice researchers in the biodiesel area.

## 2. Biodiesel Production

Biodiesel, or fatty acid alkyl ester (FFAE), is made up of methyl or ethyl esters produced from vegetable oil or animal fat. It is well-known to be an alternative sustainable fuel source to decrease the depletion rate of fossil fuels, due to its similar fuel properties to the diesel fuel. Biodiesel is a non-toxic and biodegradable fuel, which produces less harmful gases and thus reduces pollution to the environment.

Numerous kinds of feedstocks have been introduced to prepare biodiesel; for instance, refined oils [11], recycled waste cooking oils [12], and microalgae [13]. Commonly, these different types of feedstocks can be classified into three generations. The first-generation feedstock is the edible oils (vegetable oils), including soybean, palm tree, corn, coconut, rapeseed, sunflower oils, and others. Among these, the dominant feedstock is the soybean oil, while the palm tree is well-known due to its capability to produce the highest quantity of oils per cultivated area [14]. Nevertheless, vegetable oil conflict arose along with an upsurge of vegetable oil prices as its demand increased. Thus, the second-generation feedstocks that do not conflict with food interest have been presented: the oils of non-edible crops. Oils harvested from rubber seed, castor, jatropha, maize, and animal fat belong to this type. Some of these non-edible crops have been used in the production of commercial products such as cosmetics, and thus conflict arose again. Microalgae and other biodiesel feedstocks that do not conflict with the interests related to human consumption are considered to be third-generation feedstocks. Algae are predominant under this category due to their superior properties, and they bring numerous benefits. Algae have a higher oil content in comparison with other feedstocks such as sugar cane and corn. Similarly to a plant, algae consume carbon dioxide and release oxygen into the environment during their photosynthesis process. Thus they can be used to capture the carbon dioxide from the industrial flue gasses in order to reduce the air pollution. In addition to this, they have a strong adaptability to their living environment, even in polluted water, due to their unicellular or multicellular structure [15]. Furthermore, the fuels produced from algae are carbon-neutral. Different biodiesel feedstocks [16] are outlined in Table 1.

Biodiesel is environmentally friendly and thus it holds great promise in solving the environment-related problems, such as air pollution, and the greenhouse effect. Nevertheless, biodiesel production encounters one major challenge, which is the production cost in terms of its feedstock and oil-to-biodiesel conversion process. In order to reduce the cost of feedstocks, low-cost feedstocks such as palm oil and waste cooking oil have been used to produce biodiesel. The conversion process costs can also be reduced by conducting optimization and process enhancement.

Transesterification is a process in which the alcohol, usually methanol or ethanol, is used to break the triglycerides (oils or fats) into methyl or ethyl esters, the renewable biodiesels. This process can be conducted in either the presence or absence of an acid/alkali-based catalyst. Transesterification comprises three reversible reactions, Equations (1)–(3), that form diglyceride, monoglyceride, and glycerine consequently, and at the same time produce one ester molecule for each alcohol molecule consumed in each reaction step. Thus, the overall reaction, as shown in Equation (4), involves 1 mole of triglyceride and 3 moles of alcohol to form 3 moles of alkyl esters and 1 mole of glycerol as a by-product. Practically, however, an excess quantity of alcohol is added to complete the conversion process and to increase the alkyl ester (biodiesel) yields by shifting the reaction equilibrium towards the production of esters. Additionally, the excess alcohol allows the phase separation of biodiesel from the glycerol.

(1) Triglyceride (oils or fat) + ROH ⇄ Diglyceride + R′COOR

(2) Diglyceride + ROH ⇄ Monoglyceride + R′COOR

(3) Monoglyceride + ROH ⇄ Glycerol + R′COOR

(4) Triglyceride (oils or fat) + 3ROH ⇄ Glycerol + 3R′COOR

Generally, there are three types of heating methods to perform the transesterification process, which are conventional heating, supercritical heating, and microwave heating. Table 2 [14] compares and summarizes their characteristics and their respective pros and cons. Their details and mechanisms, however, will be discussed further in the later sections.

### 2.1. Catalytic Transesterification

**Figure 1:**
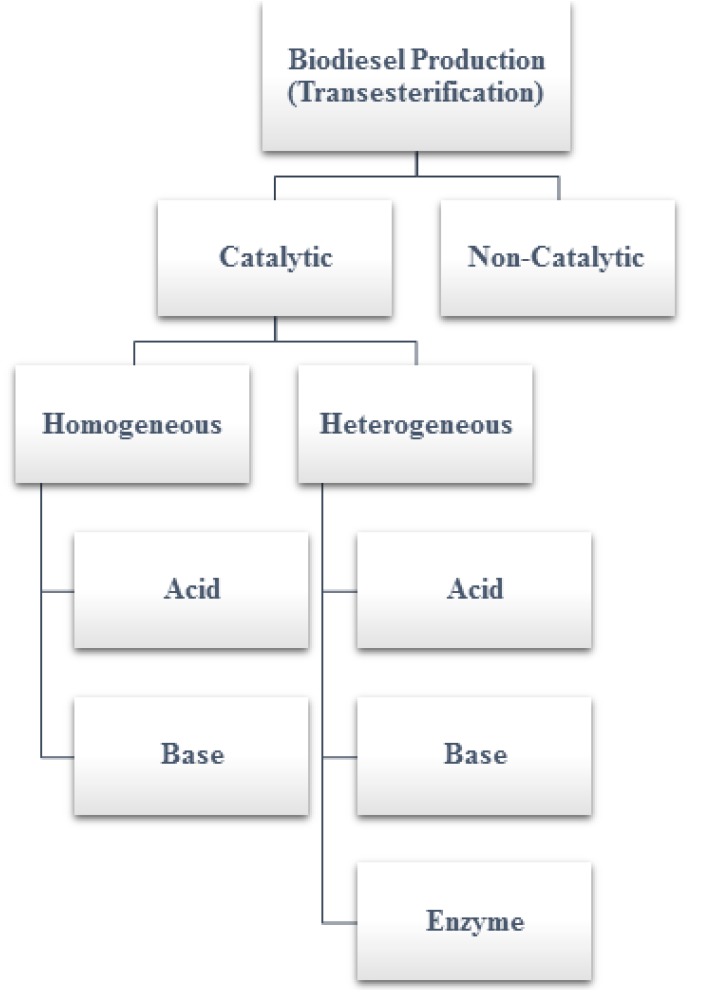
Biodiesel production classification.

As described above, transesterification can be performed with or without a catalyst. There are two major types of catalysts: the homogeneous type and the heterogeneous type. Moreover, they can be further categorized (Figure 1) into three groups: (a) acids, (b) bases, and (c) lipase enzymes. Catalysts are used to shorten the reaction time and to produce biodiesel of better quality. These catalysts are employed depending on the type and quality of the feedstock. The alkaline catalysts are usually preferable as they require a lower reaction temperature and provide a higher reaction rate and conversion efficiency when compared with the acid catalysts [17]. On the other hand, acid catalysts are insensitive to FFAs, compared to base catalysts. FFAs of the feedstock reduce the transesterification rate by reacting with the catalyst and forming unwanted soap instead of biodiesel. Hence, acid catalysts are superior for transesterification that involves vegetable oils with a higher percentage (more than 1%) of FFAs [17]. In addition, lipase enzymes are an alternative catalyst applied to accelerate the transesterification reaction. Lipase enzymes have outstanding advantages over the acid/alkali-based catalysts, as they reduce the complication of downstream processes such as product separation, purification, washing, and neutralization. They are also suitable for biodiesel feedstock with a high FFA content. Nevertheless, there are disadvantages to the lipase enzyme, such as that they are more expensive and require a longer reaction time [18].

Homogeneous-base catalysts such as sodium hydroxide (NaOH), potassium hydroxide (KOH), and sodium methoxide (CH_3_ONa) are the most common catalysts used for biodiesel production. Among these, CH_3_ONa is the most effective, as less is needed, in comparison to NaOH and KOH, in order to obtain the same catalytic effect. In addition, NaOH and KOH catalysts also form water, which leads to soap formation and slows down the reaction rate. However, CH_3_ONa is more expensive. Homogeneous-base catalytic transesterification involves the catalysts that can be dissolved in the alcohol. At the end of this reaction, these homogeneous-base catalysts will not be recovered for the reuse purpose. Additionally, problems such as a complicated separation downstream process and unwanted soap formation have encouraged researchers to discover the application of heterogenous catalysts in biodiesel production. Other than solving the problems mentioned above, heterogeneous catalysts have a higher adaptability to the cheaper feedstocks. Moreover, their downstream process is less complicated and the catalysts can be reused, hence resulting in a lower overall production cost.

All of the methods mentioned above are known as catalytic transesterifications, as catalysts or enzymes are applied to enhance the reaction rate. These catalytic processes are commonly assisted with conventional heating. The pros and cons of different types of catalysts used in transesterification for biodiesel production are summarized in Table 3 [19,20].

### 2.2. Non-Catalytic Transesterification with Supercritical Condition

In catalytic transesterification, the catalysts are not consumed, and so a complicated purification process is essential to separate them in order to collect the biodiesel. Additionally, this reaction is relatively time-consuming due to the phase separation of the oil-alcohol mixture. In order to avoid these problems, supercritical transesterification is introduced [21,22]. In the supercritical method, the temperature and pressure are maintained above the critical values of alcohol (e.g., 239 °C and 8.1 MPa, respectively) for methanol. This decreases the dielectric constant and solubility parameter of the alcohol, and thus the alcohol becomes more soluble with the oils. As a result, the transesterification reaction time is shortened significantly. Moreover, the complicated purification process is eliminated, as no catalysts are required for the supercritical method. Thus, the oil-biodiesel conversion using this method is also known as non-catalytic transesterification. Furthermore, the supercritical method has a higher adaptability with the low-cost feedstock (higher water content and FFAs) in comparison with the conventional catalytic transesterification [23]. The supercritical method is expensive, as a higher energy input is compulsory to operate at the critical temperature and pressure.

### 2.3. Microwave-Assisted Methods

Microwave-assisted methods have been developed as a promising way—that is rapid, energy-efficient, cost-saving, and environmentally friendly—to produce biodiesel. Numerous tests and optimization studies with different feedstocks have been conducted by researchers [24,25,26,27,28] in the recent years to maximize the advantages of microwaves for biodiesel production. Other than for transesterification reactions, microwave energy is also applied to assist the oil-extraction process in order to produce a greener and better quality of biodiesel [29].

#### 2.3.1. Theory of Microwaves

Microwave dielectric heating was introduced as a new heating technique in the 1970s, and eventually became a powerful method to enhance chemical reactions in almost all areas of chemistry. Gedye et al. [6], the very first group of researchers that related microwave to organic chemistry, observed that the organic reactions could be carried out at a faster rate under microwave irradiation. Since this experiment, the application of microwave irradiation has been reviewed, and they observed that the microwave heating increased the reaction rate, and the formation of side products were reduced compared to the conventional heating method [30]. These acceleration, reactivity and selectivity changes observed during microwave-assisted reactions enabled energy saving and process intensification, and could be explained through the combination of thermal and non-thermal effects of microwaves. These effects will be explained in detail in the following section.

**Figure 2:**
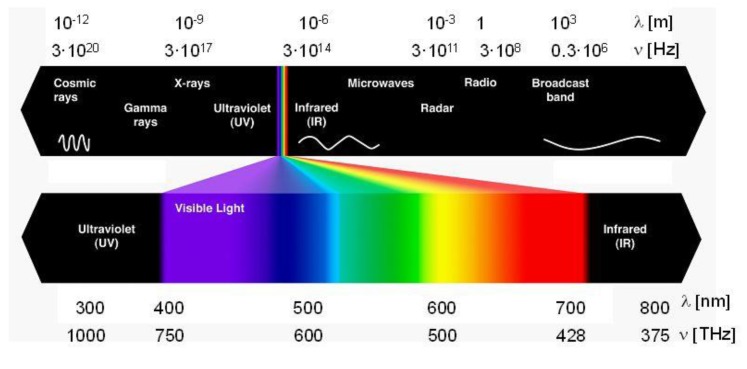
Electromagnetic spectrum [31].

Electromagnetic waves are formed by the coupling of electric and magnetic fields in such a way that they are perpendicular to each other. Microwaves are electromagnetic waves with a frequency range between 0.3 and 300 GHz, with wavelengths ranging from 0.01 m to 1 m. Microwaves are located in-between infrared waves and radio waves on the electromagnetic spectrum. Electromagnetic waves with a higher frequency are associated with higher energy and shorter wavelengths. Figure 2 shows the electromagnetic wave spectrum.

Domestic applications such as microwave ovens are approved to be operated at a frequency of 2.45 GHz, while the industrial applications are between 0.915 GHz and 2.45 GHz. Most of the reported microwave chemistry experiments are carried out at 2450 MHz [32,33,34], as the maximum microwave energy is absorbed by liquid water near to this frequency. There are numerous studies that have been carried out on microwave applications in the organic synthesis sector; for instance, the application of microwaves in the synthesis of polymer [35] and metal oxides [7], solvent free reactions [36], homogeneous and heterogeneous catalytic reactions [37], and many other reactions involved in medicinal [38] and green chemistry [8].

The quantum energy of microwaves is given by Planck’s law:

(5) E = hv

Within the frequency range of microwaves (0.3–300 GHz), respective energies associated are between 1.24 × 10^−6^ and 1.24 × 10^−3^ eV. These microwave energies are lower than the energy associated with Brownian motion. In other words, microwave energy is not sufficient to break chemical bonds and thus cannot induce chemical reactions. Table 4 gives the values of some bond energies and those of microwave radiation.

In conventional heating, heat is transferred through thermal conduction and convection, through the wall of the targeted object, to reach its internal materials. Thus, this heating method is also known as wall heating. In this case, the sample results in a non-uniform temperature and higher thermal gradients, as the heat is transferred through the other mediums before it reaches the internal sample. The heating effect is heterogeneous and it is also highly dependent on the materials’ properties, such as thermal conductivity, specific heat capacity, and density. Moreover, a large portion of energy [14] from the heat sources will be lost to the environment through conduction and convection. Hence, this is a relatively slow and unproductive way to transfer energy into the reacting system.

In contrast, microwave heating utilizes the ability of certain materials, usually solids or liquid, to transform electromagnetic energy into heat energy. The two components of microwaves (electric field and magnetic field) will interact differently with the material under different mechanisms. The electric and magnetic field components are responsible for the microwave dielectric heating and magnetic loss heating, respectively. Among these two heating effects, most of the studies reported the microwave dielectric heating effect [39], however, according to Cheng et al. [40,41], there are superior benefits of microwave magnetic-field heating for certain magnetic dielectric and conductive powder materials. Peng et al. [42] presented that the magnetic loss in the microwave heating of ferrites is up to approximately 4 times greater than the dielectric loss.

**Figure 3:**
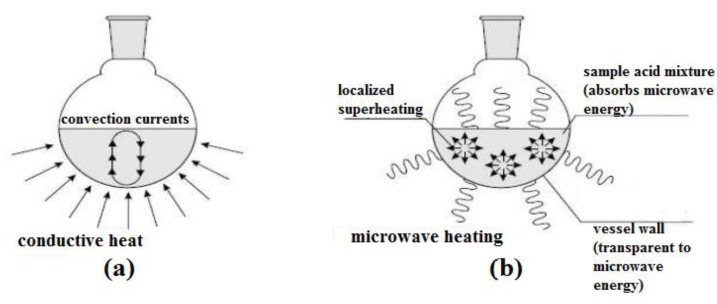
Heating mechanisms for: (**a**) conventional heating, and (**b**) microwave heating [43].

In the organic chemistry field, almost all of the microwave heating reported refers to dielectric heating. Essentially, there are two primary dielectric loss mechanisms for microwave dielectric heating: (a) dipolar polarization, and (b) ionic conduction, which lead to an instantaneous localized and rapid superheating of the reaction materials. Here, the molecules of the whole reaction mixture are coupled with the microwaves, resulting in a rapid increase in temperature. The heating of the reaction vessel is not involved in this process. Hence, microwave dielectric heating is not restricted by the thermal conductivity of the vessel, unlike the conventional heating method. However, this depends on the shape, dielectric properties and size of the reaction materials, and lastly, the nature of the microwave equipment used. For more details about the microwave heating mechanism, some excellent reviews can be found in [39] and [43]. Figure 3 illustrates the basic differences between conventional heating and microwave heating. The characteristics of microwave heating and conventional heating are also summarized in Table 5 to give an overall comparison of their features [44].

#### 2.3.2. Thermal Effect of Microwaves

Several researchers conclude that the acceleration of a reaction is only due to the thermal effect, also known as the kinetic effect. This effect is caused by the rapid increase of temperature during the dipolar polarization and/or ionic conduction under microwave irradiation [45]. According to this group of researchers, it cannot be denied that the reaction time is dramatically reduced by using microwave heating when compared to conventional heating, but the kinetic mechanism of the same reactions remains identical. They explained that the acceleration of the reaction is due to the rapid, sudden and uncontrollable increasing of the temperature under microwave irradiation.

To explain the thermal effect of microwaves, the fundamental thermodynamic formula, which is the Arrhenius equation, is applied, as there is involvement of thermodynamics of reaction materials.

(6) $$K\;=\;A\times e^{\frac{-\Delta G}{RT}}$$

where *K* is the reaction rate, *A* is a collision frequency factor that indicates the molecular mobility based on the molecular vibration frequency during the reaction, Δ*G* represents the activation energy, *R* is the gas constant, and *T* is the temperature in kelvin. Based on Equation (6), there are two possible methods to enhance the chemical reaction rate, which are by increasing the collision frequency factor, *A*, and by decreasing the activation energy, Δ*G*.

Activation energy can also be expressed in term of enthalpy, *H*, and entropy, *S*, with this equation:

Δ*G* = Δ*H* − *T*Δ*S* (7)

As shown in Equation (7), in order to decrease Δ*G*, Δ*S* (entropy) should be increased. This phenomenon can be attained in microwave-assisted reactions, as Δ*S* is higher due to the involvement of the interactions at the molecular level and also the rapid, random movement of the dipole. Additionally, activation energy can be reduced through the superheating condition achieved under microwave irradiation, as the result of the microwave energy dissipation over the whole reaction volume [46].

Some researchers [47,48] have claimed the existence of hot spots, which are certain particular areas with a higher energy and temperature, in the reaction mixture under microwave irradiation due to the inhomogeneity of the applied electromagnetic field. The non-homogenous distribution of microwave fields is created within the microwave ovens as a consequence of their reflections on the inner walls. Filter paper that is soaked with cobalt chloride solution, however, can be used to determine the location of a high-energy hotspot [43].

A hotspot usually acts as an active site to promote the catalytic activities and thus contribute to the improvement of the conversion rate and efficiency. Durka [47] concluded that for a given methanol conversion, a lower net heat input was applied to the reactor under microwave heating when compared to electric heating, due to the formation of hotspots. Moreover, Ren [49] has demonstrated this hotspot effect on the decomposition of hydrogen sulfide, H_2_S, over a molybdenum disulphide, MoS_2_, catalyst under microwave conditions. Generally, the H_2_S decomposition rate—or strictly speaking, the catalytic activity—is increased along with temperature. However, the results showed that the decomposition rate increased with the microwave power at the beginning of experiment, but that any further increment of the power supply after 100 W led to a decreasing of the decomposition rate due to the formation of hotspots where the temperatures were increased too rapidly until the active *H* and *S* may have gone through a new reacting pathway.

**Figure 4:**
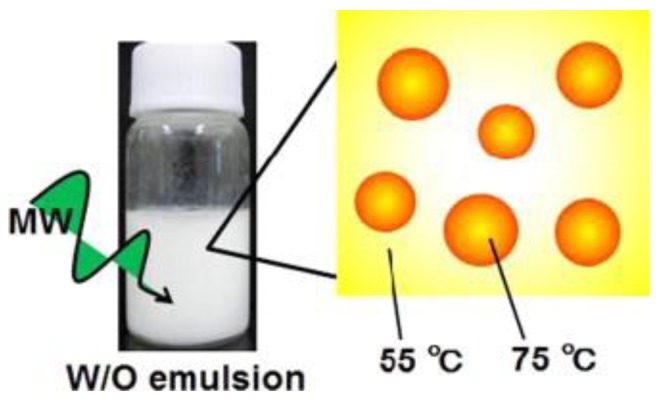
Selective heating of water/oil emulsion (water droplets are represented by the orange bubbles) [54].

**Figure 5:**
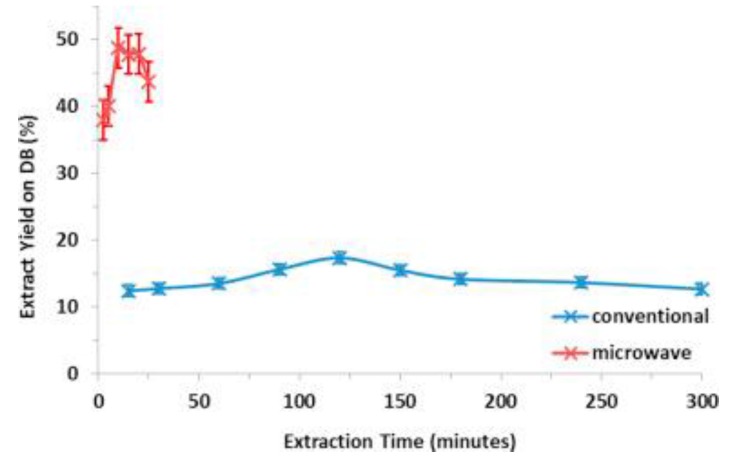
Effect of extraction time on extract yield (DB) at 70 °C [50].

Microwave irradiation is also well-known due to its selective mode of heating. Generally, all of the interactions between materials and microwaves can be categorized into three groups, which are absorption, transmittance, and reflection. Naturally, in a reaction mixture under microwave irradiation, the microwave will be absorbed by the polar substances and heat is generated within them. At the same time, the nonpolar substances of the reaction mixture that do not absorb microwaves, however, are not heated. This selective mode of heating has been utilized and studied by numerous researchers in various fields [3,50,51,52], and can be grouped into these three categories: solvents, catalysts, and reagents [53]. Takuya et al. [54] illustrated the selective heating mode of microwave irradiation through their study on the size effect of water droplets in water/oil emulsion with sorbitan fatty acid monostearate surfactant. They observed that the water droplets’ temperature was 20 °C more than the whole reaction solution, and they concluded that this phenomenon occurred due to the selective microwave heating (Figure 4). Moreover, they found that in the water/oil emulsion, water droplets with a different size and concentration result in a different selective microwave heating rate. The application of selective microwave heating in order to enhance the extraction rate and yield was presented by Lee et al. [50]. A step-change of extraction yield was detected at the temperature for which the selective heating of the biomass, in this case the okra, occurred. Also, due to the principle of microwave selective heating, the extraction rate of okra with microwave heating was significantly higher, and at the same time produced a higher yield in comparison with the conventional method (Figure 5). Satoshi and his co-workers [55] emphasized that the dehydrogenation of organic hydride methylcyclohexane was at a faster rate and gave a higher yield as the result of the selective heating of the palladium/activated carbon (Pd/AC) catalyst under microwave irradiation. The Pd/AC catalyst was heated to 340 °C within 2 min, while the conventional heating took 35 min to reach a temperature at which the catalyst started to work efficiently.

#### 2.3.3. Non-Thermal Effect of Microwaves

Some authors [56,57] claimed that the thermal effect alone is not sufficient to explain microwave results such as reaction rate enhancement and thus eventually they postulated the existence of non-thermal microwave effects. Even today, this issue remains a controversial topic among researchers [58,59]. It has been argued that microwave irradiation affects the pre-exponential factor or activation energy in the Arrhenius equation, *A*, due to the orientation effects of dipolar molecules in the presence of an electric field. In polar reaction mechanisms also, the polarity is increased under microwave irradiation, from the ground state to the transition state. This eventually causes the activation energy to be decreased and results in an enhancement of reactivity. This statement has been supported by the work of Xu et al. [60].

To prove the existence of microwave non-thermal effects, a model has been proposed by Zhou et al. [61] to study the reason for the reduction in the apparent activation energy, *Ea′*, under microwave irradiation, which results in an accelerating of chemical reactions. This model follows four hypotheses: (1) Matter is ideal and uniform. (2) A portion of microwave energy is absorbed by the molecules, and interacts with the molecular matter directly to reduce the *Ea′*, illustrating a vital microwave catalytic effect. (3) At the same time, another portion of microwave energy is released on the molecular matter by the thermal effect, causing the increasing of the reaction system’s temperature. (4) A dynamic balance between the absorbed and released microwave “energy molecular” on the molecular matter will be achieved under balanced circumstances.

Eventually, a formula that is used to estimate quantitatively the reduction of *Ea′* due to the microwave energy was established successfully:

(8) $$Ea^{\prime }\;=\;Ea_{0}\;-\;Ea_{MW}$$

where *Ea*_0_ represents the activation energy barrier of the reaction system without any microwave irradiation, and *Ea_MW_* is the portion of microwave energy that is transformed to reduce the *Ea′* directly.

*Ea_MW_* can also be expressed as Equation (9), in terms of the microwave electromagnetic field (*E_MW_*), microwave energy power, (*P_MW_*), the total microwave energy absorbed by the matter (*E_t_*) and also a constant (*k*) that is determined by the microwave frequency and the dielectric properties of the reacting system. The details of the equation’s development can be found in [61].

(9) $$Ea_{MW}\;=\;\frac{kE_{MW}P_{MW}}{1\;+\;kE_{MW}P_{MW}}\;E_{t}$$

**Figure 6:**
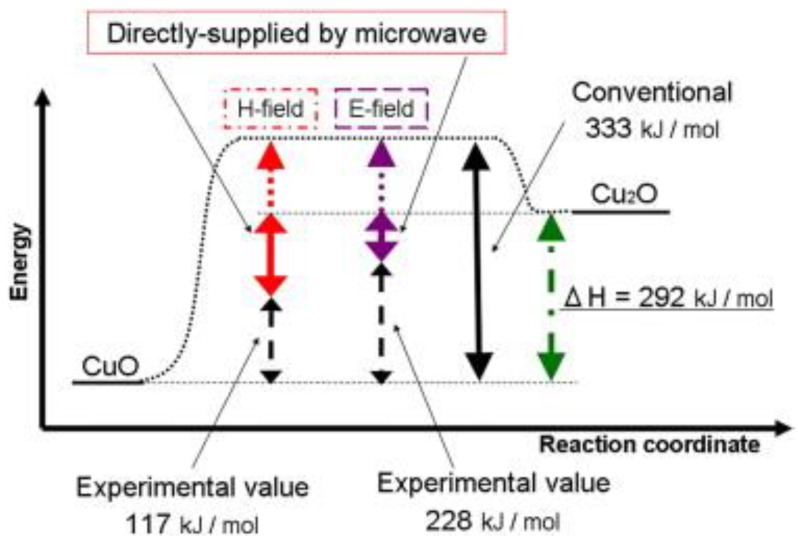
Reaction coordinates of CuO reduction under conventional heating, *E*-field microwave heating, and *H*-field microwave heating.

In addition, there is some other evidence regarding the existence of a microwave non-thermal effect. For instance, Fukushima et al. [62] investigated the behavior of copper(II) oxide reduction under microwave irradiation, and the results suggested that the reduction energy was supplied by both thermal energy and microwave energy (magnetic, *H*, and electric, *E*, fields) as indicated in Figure 6. Strictly speaking, microwave energy could transform directly into reduction energy in the energy dissipation process by interacting directly with the valence electrons. Thus, they concluded that the reduction of copper(II) oxide involved thermal and non-thermal process.

Moreover, Mao et al. [63] reported that the synthesis of quaternary ammonium salt with the microwave-assisted process was far more effective due to its lower activation energy (41.44 kcal/mol) in comparison to the conventional thermal heating method (61.21 kcal/mol). This phenomenon was explained as the result of the non-thermal microwave effect. Furthermore, a sign of the non-thermal microwave effect was also observed by Nazari and Ghandi [57] in their study on oxidation of aromatic α-diketones. The activation energy and pre-exponential factor for microwave-assisted reactions were significantly changed when compared to the conventional oil bath method. This consequently resulted in larger rate constants. Since the experiments were conducted under similar conditions, other than in the presence of microwave irradiation, rapid heating (thermal effect) solely was not adequate to explain the outcomes of this study, and thus the existence of non-thermal effects was claimed.

Other than the reaction rate enhancement, there is further evidence of a non-thermal microwave effect, which is the alternation of electric conductivity in an aqueous solution. This effect was clearly detected in a sodium chloride, NaCl, aqueous solution under a microwave field with the improved experimental set-up by Tian and Huang [64]. They provided a direct indication of the presence of non-thermal microwave effects.

**Figure 7:**
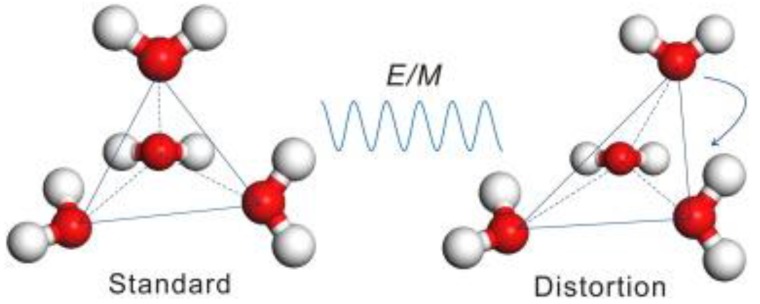
Schematic illustration of the tetrahedral structure of water before and after microwave irradiation.

**Figure 8:**
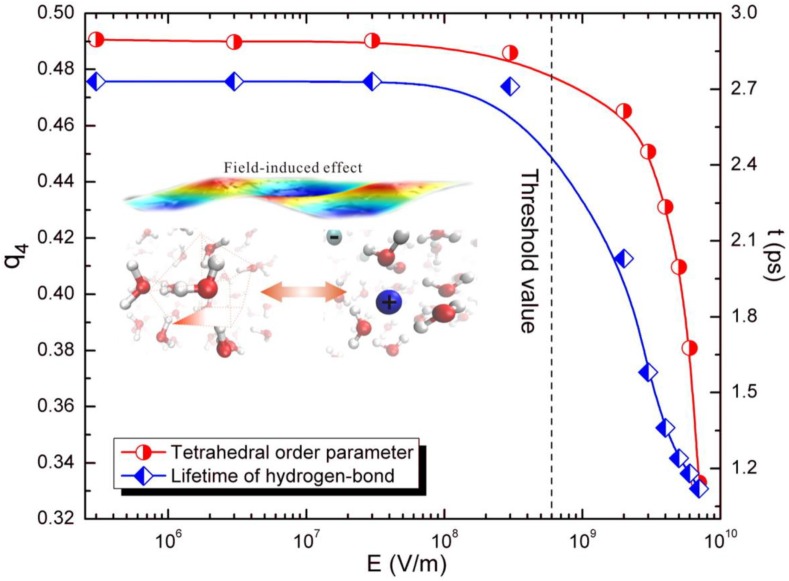
The field dependence of the tetrahedral order parameter and lifetime of hydrogen bonds.

Liu et al. [65], however, proved the existence of non-thermal effects from the perspective of molecular dynamics. The results (Figure 7 and Figure 8) showed that the dynamics of the water structure did not alter monotonically with the variation of the microwave field strength, but a threshold effect was observed. Once the microwave field strength was greater than the threshold value, a significant distortion of the water structure was spotted. Moreover, there was an abnormal pattern of the microwave-electrolyte interaction due to the rapid increases of polarizability, and the microwave field changed the system structure.

#### 2.3.4. Recent Study of Microwave-Assisted Biodiesel Production

**Figure 9:**
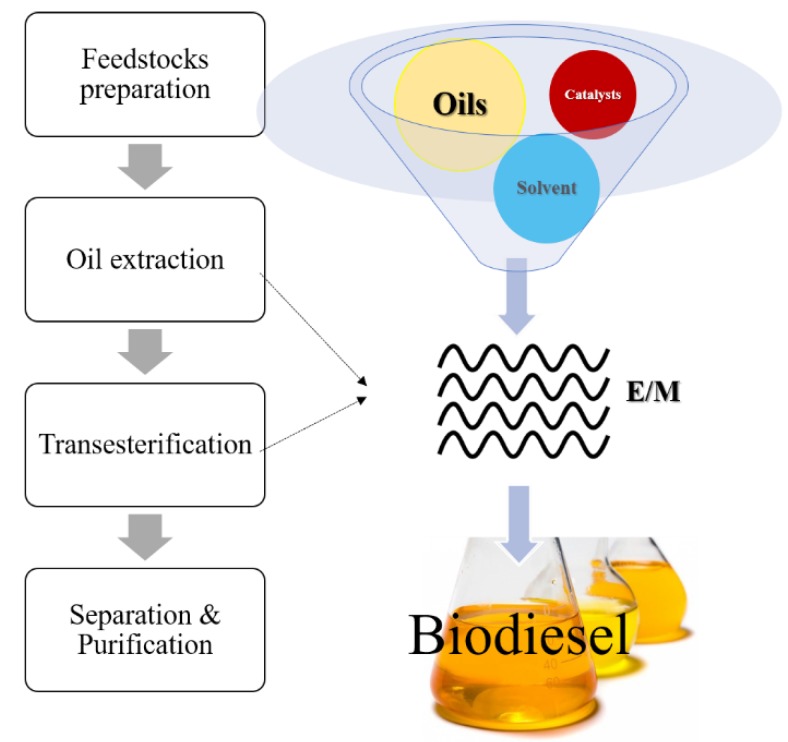
Microwave-assisted catalytic biodiesel production.

Microwave-based biodiesel production (see Figure 9) usually starts with the mixing process of oil, alcohol, and a catalyst in the appropriate ratio. Then, the mixture is processed under microwave irradiation. Lastly, separation and purification are carried out to yield biodiesel. In microwave-assisted catalytic transesterification, methanol is mostly used due to its high capability to absorb microwaves, and it is also a high-polarity organic solvent. Hence, since solvents with a high dielectric loss factor are used, the transesterification process can be improved through dipolar polarization and ionic conduction under microwave irradiation, which has a higher dependency on the polarity and absorption capability of the reacting samples. The experimental studies of microwave-assisted transesterification with different feedstocks are summarized in Table 6.

Based on the study of Zahir et al. [74], it was proved that microwave irradiation has superior benefits over the conventional heating method in biodiesel preparation. Under microwave irradiation, the reaction time has reduced from 2 h to 6 min, and at the same time it produces a higher biodiesel conversion yield (4.1% higher than the conventional method). Moreover, the energy consumption for microwave heating (288 kJ) was reported to be lower than for the conventional heating (3150 kJ) by Patil and his co-worker [24] in their experiment on biodiesel production using sulfuric acid. Other than enhancing the reaction rate, producing higher yield, and consuming less energy, microwave-assisted transesterification produces less by-product, which results in a simplified downstream process and eventually reduces the time taken for product separation [14,24].

There are two major biodiesel standards that have been followed by people throughout the world, which are the American Standard Specification for Biodiesel (ASTM 6751), and the European Standard for Biodiesel (EN 14214). Microwave-based biodiesel properties were very well compared with these standards by several researchers, and are recorded in Table 7.

**Figure 10:**
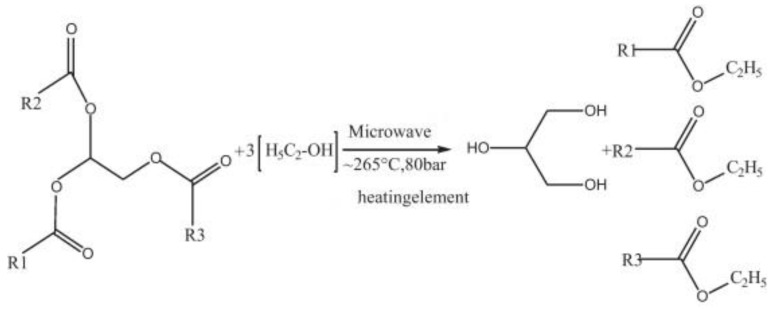
The microwave-mediated transesterification of algal biomass under supercritical ethanol conditions to yield ethyl ester.

**Figure 11:**
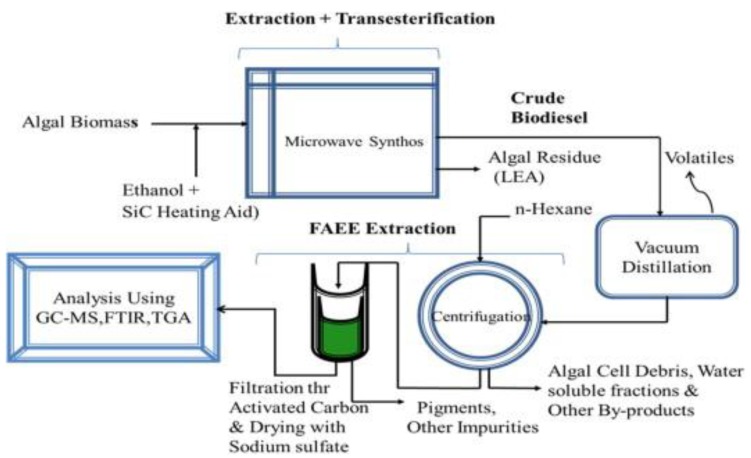
Experimental protocol for the microwave-mediated supercritical ethanol transesterification process for algal biomass.

Furthermore, microwave energy can also be applied to enhance the supercritical reaction for the non-catalytic transesterification, especially for the feedstocks that contain water [14]. This is because water molecules consist of a dipole moment that is sensitive to the electric field. Under microwave irradiation, a dipole will try to align itself with the electric field applied by rotation, and as a result, local superheating is formed. Patil P. et al., demonstrated the direct conversion of dry [80] and wet [81] algal biomass (*Nannochloropsis salina*) into biodiesel with a non-catalytic transesterification method under microwave-mediated supercritical ethanol conditions (see Figure 10 and Figure 11). The results showed that the algal extraction had been improved dramatically. Moreover, this approach also increased the reaction efficiency and production yield, and at the same time, the extractive-transesterification time had been reduced. In addition, this technique resulted in highly purified and harmless products, and thus its separation and purification stages were less complicated. From there, the energy consumption and overall production cost of microwave-assisted reactions may be reduced in comparison to the conventional heating methods.

#### 2.3.5. Future Outlook on Microwave Application in Biodiesel Production

As mentioned previously, transesterification can be performed with or without the presence of catalysts. The catalytic transesterification offers a high biodiesel conversion yield, however, it has several drawbacks, such as a complicated purification process, a low adaptability to cheaper feedstocks, and a large volume of waste water generated. To minimize these problems, non-catalytic transesterification has been proposed. Until now, non-catalytic biodiesel conversion methods have been limited to the use of supercritical conditions (i.e., temperatures above 350 °C and pressures above 15 MPa). In spite of the numerous advantages, the operational and equipment costs for producing biodiesel under supercritical conditions are huge obstacles. In this regard, non-catalytic biodiesel conversion with the conditions at temperatures around 250 °C and under ambient pressure (around 1 MPa) would be an alternative pathway to be explored. The non-catalytic transformation of lipids to biodiesel can be further justified as an economically viable process due to the high purity of glycerol that can be produced via this technology.

As described in this paper, numerous studies have been conducted to fully utilize the microwave energy for catalytic transesterification. Nevertheless, only very few studies focused on the implementation of microwaves into non-catalytic transesterification under supercritical conditions. However, both of the cases proved that microwave heating is a better heating method when compared to conventional heating, in terms of the reaction time, production yield, energy consumption, and so on. Hence, it is believed that the non-catalytic transesterification reactions at milder conditions can be achieved through the implementation of microwave energy. This technology has not been reported so far. Therefore, systematic and mechanistic investigation on the green process of microwave-assisted, catalyst-free transesterification under ambient conditions for biodiesel production is needed to be explored.

## 3. Conclusions

It is evident that the microwave-assisted reaction is extremely beneficial, in terms of both the reaction rate and energy consumption. Several reactions that do not occur under conventional heating can be achieved with high yields and better quality under microwave irradiation. These superior microwave results can be explained by the thermal and non-thermal effects of microwave irradiation, which cannot be reproduced easily by the conventional methods. The thermal effect refers to the outcome caused by the dielectric heating, which brings differences in the temperature regime, such as superheating, hotspot formation, and selective heating. The non-thermal effect, however, is defined as the specific radiation effect that is not caused by different temperature regimes, but is due to the non-thermal interactions between the substrates and microwave irradiation.

Besides the theoretical parts of microwave energy, this paper also highlights its great potential in biodiesel production. Numerous types of feedstocks have been introduced to prepare biodiesel: vegetable oil, non-edible oil, waste cooking oil, and algae. With the assistance of microwave technology, the advantages of microwaves have been brought over to biodiesel production. A rapid, energy-efficient and environmentally friendly way to produce biodiesel has been developed under microwave heating. The conversion efficiency and yield show that microwaves have the potential for large-scale biodiesel production, as they able to interact with various components. Furthermore, the non-catalytic transesterification at subcritical conditions (<300 °C, 1 bar) instead of supercritical conditions (>350 °C, 15 bar), with the assistance of microwave energy, should be developed in order to solve the cost problem of the conventional non-catalytic transesterification due to its higher energy input to achieve the supercritical condition.

It is undeniable that most of the results collected so far are from the laboratory-scale experiments, and so the reproducibility of similar outcomes at the industry level is still doubtful. Nevertheless, it seems very plausible that the biodiesels used in the future may involve microwaves at some points in their production processes. Certainly, more in-depth research is necessary for the scaling-up purpose in terms of the process design and optimization, the reaction kinetics and thermodynamics, and the protocols of biodiesel analysis.

## Figures and Tables

**Table 1:** Different feedstocks for biodiesel production.

Vegetable/Edible Oils	Non-Edible Oils	Animal Fats	Other Feedstocks
Soybeans	Almond	Lard	Bacteria
Rapeseed	*Abutilon muticum*	Tallow	Algae
Canola	Andiroba	Poultry Fat	Fungi
Safflower	Babassu	Fish oil	Microalgae
Barley	*Brassica carinata*		Tarpenes
Coconut	*B. napus*		Latexes
Copra	Camelina		Cooking oil (Yellow Grease)
Cotton seed	Cumaru		Microalgae (Chlorellavulgaris)
Groundnut	*Cynara cardunculus*		
Oat	*Jatropha curcas*		
Rice	*Jatropha nana*		
Sorghum	Jojoba oil		
Wheat	Pongamiaglabra		
Winter rapeseed oil	Laurel		
	Lesquerellafendleri		
	Mahua		
	Piqui		
	Palm		
	Karang		
	Tobacco seed		
	Rubber plant		
	Rice bran		
	Sesame		
	Salmon oil		

**Table 2:** Comparison between three types of heating methods for biodiesel production.

Characteristic/Parameter	Conventional Heating	Supercritical Heating	Microwave Heating
Reaction time	1 to 2 h (Long)	Less than 1 h (Short)	0.05 to 0.1 h (Very short)
Reaction temperature	40 °C to 100 °C	250 °C to 400 °C	40 °C to 100 °C
Reaction pressure	Atmospheric	35 MPa to 60 MPa (High)	Atmospheric *
Catalyst required	Yes	No	Yes/No
Heat losses	High	Moderate	Low
Energy form	Electrical energy to thermal energy	Electrical energy to thermal energy	Electrical energy applied through microwave
Process efficiency	Low	Moderate	High
Catalyst removal	Yes	No	Yes
Soap removal	Yes	No	Yes
Advantages	Simple to operate, uses low energy source	Short reaction time, easy product separation	Short reaction time, cleaner products, energy efficient
Limitations	High energy requirement, saponified products	High capital costs, pressure vessel safety	May not be efficient with feedstock containing solids

* Reactions at high temperature and pressure with no catalysts are possible.

**Table 3:** Advantages and disadvantages of different types of catalysts in biodiesel production.

Type of Catalyst	Advantages	Disadvantages
Homogeneous base catalyst	Very fast reaction rate: around 4000 times faster than acid-catalyzed transesterification. Reaction can be carried out at mild reaction conditions (less energy intensive). Catalysts such as NaOH and KOH are relatively cheap and widely available.	Sensitive to free fatty acid (FFA) content in the oil. Unwanted soap formation if the FFA percentage in the feedstock is more than 2 wt %. Decrease in the biodiesel yield due to soap formation. The downstream purification process raises problems such as producing a large amount of wastewater.
Heterogeneous base catalyst	Relatively fast reaction rate in comparison to acid-catalyzed transesterification. Reaction can be carried out at mild reaction conditions (less energy intensive). Easy separation of catalyst after the reaction. High possibility to reuse and regenerate the catalyst.	Poisoning of catalyst when exposed to the surrounding air. Sensitive to FFA content in the oil due to its basicity property. Unwanted soap formation if the FFA percentage in the feedstock is more than 2 wt %. Decrease in the biodiesel yield due to soap formation. Leaching of catalyst active sites may lead to product contamination.
Homogeneous acid catalyst	Insensitive to FFAs and water content in the feedstock. Preferable method for cheaper feedstock. Simultaneous esterification and transesterification processes. Reaction can be carried out at mild reaction conditions (less energy intensive).	Very slow reaction rate. Corrosive catalyst such as H_2_SO_4_ leads to corrosion on reactor and pipelines. Separation of catalyst from product is problematic.
Heterogeneous acid catalyst	Insensitive to FFA and water content in the feedstock. Preferable method for cheaper feedstock. Simultaneous esterification and transesterification processes. Easy separation of catalyst from the product. High possibility to reuse and regenerate the catalyst.	Complicated catalyst synthesis process results in higher overall production cost. Normally, a high reaction temperature, high alcohol-to-oil molar ratio, and long reaction time are required. Energy intensive. Leaching of catalyst active sites may lead to product contamination.
Enzymes	FFAs are converted to biodiesel. Not affected by the water content in the feedstock. High biodiesel yield, usually around 90%. Simple glycerol recovery and produces high grade glycerol. Easy catalyst recovery and the reusability proved but not sufficiently studied. Low reaction temperature at 20–50 °C. Low environmental impact as no need for wastewater treatment.	Low to moderately-high reaction rate, depending on the parameters. Relatively high cost of catalysts if enzymes cannot be recovered and reused. Possible enzyme inhibition by alcohols.

**Table 4:** Energy of different bonds and microwaves.

Bonding and Microwave	Energy (eV)
Brownian motion at 37 °C	2.7 × 10^−3^
Biological compound	13.6
Covalent bond (e.g., OH^−^)	5
Hydrogen bond	2
Van der Waals intermolecular interactions	<2
Microwave at 0.3 GHz	1.24 × 10^−6^
Microwave at 2.45 GHz	1 × 10^−5^
Microwave at 300 GHz	1.24 × 10^−3^

**Table 5:** Comparison between microwave heating and conventional heating.

Microwave Heating	Conventional Heating
Energetic coupling	Conduction/convection
Coupling at molecular level	Superficial/wall heating
Rapid	Slow
Volumetric	Superficial
Selective	Non-selective
Dependent on material’s properties	Less dependent

**Table 6:** Experimental studies of microwave-assisted transesterification with different feedstocks.

Feedstock	Feedstock Solvent	Reaction Time (min)	Reaction Temperature (°C)	Catalysts	Microwave Power (W)	Biodiesel Yield (%)	Reference
Coconut oil	1:6 Methanol	5		1% NaOH	100	97.76	[66]
Waste frying oil	1:6 Methanol	2	60	1% NaOCH_3_		98.87	[27]
	1:12 Methanol	6		2% BaO	800	96	[24]
	1:9 Methanol	6		2% KOH	800	92	[24]
Chinese tallow tree	1:3 Methanol (*w/v*) (co-solvent: hexane)	20	58.1	1.74% NaOH		96.62	[67]
Palm oil	1:12 Methanol	1.75	70	1% NaOH	400	99.4	[68]
	1:5 Methanol	10		0.5% Ca(OH)_2_ from seashells		96	[69]
	1:9 Methanol	60	65	5% CaO	150	89.9	[37]
Soybean oil	1:12 Methanol	2	70	1% KOH	200	99	[70]
	1:14 Methanol	30	65	10% C_4_H_4_O_6_KNa doped ZrO_2_ catalyst		94.75	[28]
Castor oil	1:10 Ethanol	10	60	1.5% KOH		80.1	[26]
*Pongamia pinnata*	1:6 Methanol	5	60	0.5% NaOH		96	[71]
	1:6 Methanol	5	60	1% KOH		96	[71]
Wet microalgae	1 g : 4 mL Methanol (co-solvents: chloroform, sulfuric acid)	30	60		400	11% of dry-mass	[21]
Microalgae oil	1:1 Methanol-hexane (*v/v*)	10	65	NaOH		86.41	[72]
Rice bran oil	1:5 Methanol	20	60	0.15% NaOH		98	[73]
Safflower oil	1:10 Methanol	6	60	1% NaOH	300	98.4	[74]
Canola	1:1 Methanol (*w/w*)	5	100	1% ZnO/La_2_O_2_CO_3_		95	[75]
Rubber seed oil	1:5 Methanol	60	60	6% Cement clinker catalyst		96.8	[76]
Yellow horn seed oil	1:6 Methanol	120	50	8% immobilized Novozym 435 (in green deep eutectic solvent)	400	95	[77]

**Table 7:** Microwave-based biodiesel properties.

Property	Units	ASTM D6751	EN 14214	Microwave Heating					Regular Diesel [78]
				Waste Cooking Oil [24]	Chinese Tallow Tree [67]	Karanja [79]	Soybean [70]	Jatropha [78]	
Density	kg/m^3^ at 15 °C	870–890	860–900	870–880	880	887	877	889	846
Viscosity	mm^2^/s at 40 °C	1.9–6	3.5–5	2.25–3.10	2.02	4.3	4.22	4.21	2.28
Pour point	°C			−4 to −1	2			6	−15
Flash point	°C	>93	>101			145	173	132	68
Cetane index		>47	>51	55.45–56.10	62.73	56.3	50.9		
Copper strip corrosion index		No. 3 max	Class 1			No. 2		No. 1	No. 1
Iodine value	g I_2_/100 g		<120			83	115.3		
Heating value	MJ/kg		32.5–36.1	45.08–45.24				37.95	42.71
Saponification value	mg KOH/g					195	181.3		
Acid value	Mg KOH/g	<0.5	<0.5		0.34	0.405	0.14	0.42	0.34
Water content	%	<0.05	<0.05		6.00%		2.00%	0.0129	0.0102
